# When to start ART in Africa – primarily guided by RCTs or patient autonomy?

**DOI:** 10.7448/IAS.16.1.18756

**Published:** 2013-07-12

**Authors:** Wim Delva, Yvette Fleming, Lois Chingandu

**Affiliations:** 1 The South African Department of Science and Technology/National Research Foundation (DST/NRF), Centre of Excellence in Epidemiological Modelling and Analysis (SACEMA), Stellenbosch University, Stellenbosch, South Africa; 2 International Centre for Reproductive Health, Ghent University, Gent, Belgium; 3 Stop AIDS Now!, Amsterdam, The Netherlands; 4 Southern Africa HIV & AIDS Information Dissemination Service (SAfAIDS), Harare, Zimbabwe

In a recent perspective article, leading scientists from the Centers for Disease Control and Prevention (CDC) and the International Center for AIDS Care and Treatment Programs (ICAP) argue that more data on the individual-level health benefits and risks associated with immediate initiation of antiretroviral therapy (ART) in Africa are urgently needed, before new guidelines for when to start ART in Africa can be issued [1]. To “definitively” settle the question of when to start ART in Africa, De Cock and El-Sadr propose a “large, simple” randomized controlled trial (RCT) that would assess the health risks and benefits of immediate versus deferred ART, with key end points that include tuberculosis incidence, hospitalization and death.

We profoundly disagree with this opinion and argue that inaction while waiting for the results of such a trial is unjustified. While we do not oppose an African RCT to better understand the health benefits and risks of earlier ART initiation, we make the case for a fundamental shift in thinking around ART initiation in Africa, centred on the patient's right to decide when to start ART, in consultation with his or her health care providers, and guided by *all* scientific evidence, including that from past, on-going and planned implementation studies.

In sub-Saharan Africa, ART initiation is generally not recommended in people with more than 350 CD4+ cells/µL unless they are co-infected with TB; but some countries have expanded ART guidelines to include ART initiation irrespective of CD4+ count for serodiscordant couples (Zambia and Nigeria), for HIV-positive partners of HIV-negative pregnant women (Burundi), and for HIV-positive pregnant and breastfeeding women (Malawi, Uganda and Zambia). In the United States, national ART guidelines now recommend ART initiation irrespective of CD4+ cell count. In Europe and several countries in South America, including Brazil, guidelines stipulate that ART should be offered to those whose CD4+ cell count is less than 500/µL [2,3]. The 2013 World Health Organization's (WHO) Consolidated guidelines on the use of antiretroviral drugs for treating and preventing HIV infection stipulate that ART initiation is recommended in all individuals with a CD4+ cell count of 500 cells/μL or less (but giving priority to those with advanced clinical disease or a CD4+ cell count less than 350 cells/μL); and at any CD4+ cell count in those with active TB, Hepatitis B infection and severe chronic liver disease, in HIV-positive partners in serodiscordant couples, and in pregnant and breastfeeding women.

De Cock and El-Sadr argue that this “diversity in guidelines and practice reflects a lack of definitive data indicating what is best for those who would be taking the drugs.” They further claim that conflicting evidence from observational studies, absence of data from sub-Saharan Africa and limited data from randomized trials necessitate the proposed RCT.

Of course, there is scope for refining our understanding of the risks and benefits of immediate ART initiation, but we would argue that the diversity in guidelines primarily reflects differences in the availability of financial resources across regions, as well as inter-regional differences in countries’ readiness and ability to respond to recent science on the epidemiological and health economic implications of earlier ART initiation.

Current understanding of the biological effects of HIV viral replication and ART, together with an ever-growing evidence base from (mainly) observational studies, suggest that the sooner one starts ART, the greater the reduction in morbidity and mortality in individuals living with HIV [4–7]. More empirical evidence will become available in the next one to two years from on-going studies of immediate versus deferred ART initiation in sub-Saharan Africa. While some of these are primarily designed to estimate the community-level impact of earlier ART on HIV incidence, The TEMPRANO RCT was designed to estimate individual-level benefits and risk associated with early ART initiation [8]. To wait for additional evidence from an African RCT that is yet to be designed and conducted, while observing how other regions have moved to recommending ART initiation irrespective of CD4+ count, based on the available evidence and well in advance of the results from the START and TEMPRANO trials, seems inconsistent and ethically questionable.

Moreover, in a recent meta-analysis of studies that assessed the effect of ART on TB incidence in developing countries, including the HPTN 052 RCT (where 54% of participants were from sub-Saharan Africa), ART was strongly associated with a reduction in tuberculosis incidence in adults with CD4+ counts above 350 cells/µL, with no evidence for heterogeneity of effect across the three studies [9]. New data on the CD4+ cell count trajectory during the first four years after ART initiation show that the odds of CD4+ cell recovery to 900 or more cells/µL within four years after ART initiation decreases with 10% for each incremental month of delay between the estimated date of infection and ART initiation [7] and that ART initiation during primary HIV infection can delay disease progression [6].

RCTs are most appropriate for investigating efficacy and safety of new regimens in a well-controlled environment where neither costs nor efforts are spared to achieve minimal loss to follow-up. In contrast, the variables to be assessed when deciding on national or regional ART initiation guidelines are large in number, often ill-defined or hard to measure, and necessarily include indicators of real-life acceptability, feasibility, affordability and scalability of the ART initiation policy under consideration.

More urgent than an African RCT, are implementation studies that document how offering immediate access to ART initiation, accompanied by additional investments in primary health care and community-based support, correlate with changes in HIV testing behaviour, linkage to care, treatment adherence and retention in care. Furthermore, such studies could investigate whether immediate access to ART can help to simplify treatment protocols, contribute to the elimination of new HIV infections among children, improve economic productivity and reduce the cost of pre-ART care [10].

We argue that the evidence base is already sufficient to support a global recommendation for immediate access to ART, irrespective of CD4+ cell count. However, our central tenet remains that the decision when to start ART should be made individually by each person living with HIV. Patient-centred health care does not only mean that patients’ health and wellbeing is central in the medical decision-making process but it also means that patients are granted the right to make an informed choice about if/when they want to start treatment, even if part of this information is that the benefits and the risks for individual patients are still unclear at the moment. Patient-readiness to start ART also requires that health care providers and community-based organisations support patients and communities with ART literacy and preparedness skills. We welcome the new WHO guidelines and see these as a key step in the direction of offering immediate treatment to all. It will now be up to individual countries (Governments, People Living with HIV and Civil Society) to make informed decisions about when to start treatment, taking into account the entire evidence base and human rights considerations, and not primarily base decisions on RCT data or the (perceived) lack thereof.
